# The importance of TP53/p53 in regulating the mitophagy-lysosomal machinery in muscle following disuse

**DOI:** 10.1080/27694127.2022.2047265

**Published:** 2022-03-29

**Authors:** Jonathan M. Memme, Ashley N. Oliveira, David A. Hood

**Affiliations:** a Muscle Health Research Centre; b School of Kinesiology and Health Science, York University, Toronto, Ontario, M3J 1P3, Canada

## Abstract

Since being discovered in the late 1990s, TP53/TRP53/p53 has been a focal point of research, lauded for its role as a tumor-suppressor protein. Considerable attention has also been devoted to understanding how TP53 regulates metabolic programming of the cell, focusing on the control of skeletal muscle mitochondria. This research has implications that extend not only to muscle fitness, but also to healthy aging, and the prevention of metabolic disease. Previous work from our lab and others has indicated that TP53 is involved in mediating mitochondrial quality control mechanisms in muscle, such as mitochondrial biogenesis, and clearance via mitophagy. However, the degree to which TP53 is required to regulate these mitochondrial adaptations, at this point, has remained controversial, and has not yet been studied under conditions that promote accelerated mitochondrial dysfunction, such as with prolonged muscle disuse. We set out to determine the necessity of TP53 to maintain mitochondrial content and function in muscle, both basally as well as following acute and chronic muscle disuse. To accomplish this, we made use of RNA-sequencing, among other biochemical assessments of mitochondrial content and function, whereby we assessed the effect of TP53 ablation in muscle on >20,000 genes. We provide convincing evidence suggesting that TP53 has a minor role in the basal maintenance of mitochondria in muscle; however, under the stress of chronic disuse, the absence of TP53 contributes to exacerbated declines in mitochondrial function brought about by defects in mitochondrial quality control (MQC) pathways, particularly within the mitophagy-lysosomal machinery.

We have addressed a decades-long debate about the function of TP53 (the mouse protein is TRP53, but we use TP53 herein for simplicity) in muscle metabolic health, and shown that the importance of TP53 is most evident under stress conditions where preservation of mitochondrial function is essential [1]. Whereas TP53 has been implicated in the maintenance of muscle mass during periods of muscle inactivity, its role in mediating mitochondrial content and function has not been experimentally evaluated under such conditions. Thus, we sought to determine the function of TP53 in regulating mitochondrial adaptations following acute (1 day) and chronic (7 days) sciatic nerve denervation-induced muscle disuse. We chose this model for many reasons. First, denervation elicits rapid muscle atrophy and mitochondrial decay, thus providing an accelerated model of muscle decline that would typically be observed with prolonged sedentarism or inactivity, which is pervasive in many developed societies. Second, understanding the molecular mechanisms of denervation atrophy is also translationally relevant to peripheral neuropathic conditions such as nerve injury, amyotrophic lateral sclerosis and aging itself. Third, denervation induces a robust induction of gene expression signatures relevant to mitochondrial turnover, which allows for improved analytical power in studying the disuse response in muscle.

Our data reveal that *tp53* muscle-specific knockout (mKO) animals experience similar levels of muscle atrophy as wild-type mice, which differs from a previous report that showed TP53 ablation preserves muscle mass following a shorter term (3 days) of hindlimb immobilization. However, it is worthwhile to consider the differences in both duration and intensity (denervation vs immobilization) of these disuse stimuli. This may suggest that the intensity of our disuse intervention exceeds the influence of TP53 in attenuating atrophy, and we cannot rule out the possibility that TP53 is sufficient to preserve muscle mass during shorter periods of disuse.

Principal component analysis revealed the potency of chronic denervation in altering the transcript profile of muscle, irrespective of genotype. In contrast, TP53 ablation alone has considerably less influence in accounting for the variability in gene expression, suggesting that TP53 is perhaps less important in regulating basal mitochondrial function than previously thought. However, we evaluated the interaction of denervation with genotype, which addressed the following question: are the mitochondrial and gene expression responses to denervation different in the absence of TP53? This analysis revealed that most of the downregulated gene sets are specifically related to autophagy-mitophagy, mitochondrial assembly, and oxidation/oxidative phosphorylation. When considering pathway enrichment based on all differentially expressed genes, ~60% of the most significantly affected pathways are likewise related to mitochondrial regulation. Moreover, when assessing the most affected pathways based on related clusters, we observed that “mitochondrial gene expression,” “lysosomal transport,” and “regulation of macroautophagy” are unique to the interaction term, whereas discrepant patterns of other MQC-related pathways are evident when comparing the clusters within WT and mKO denervated muscle individually. Thus, denervation is a potent stimulus for eliciting mitochondrial decline in muscle via decreased mitochondrial biogenesis, along with substantial induction of stress responses such as the mitochondrial unfolded protein response and the mitophagy-lysosome systems. These responses are attenuated in *tp53* mKO muscle, leading to a greater accumulation of dysfunctional organelles as indicated by exacerbated ROS emissions (Fig. 1).

**Figure 1.**
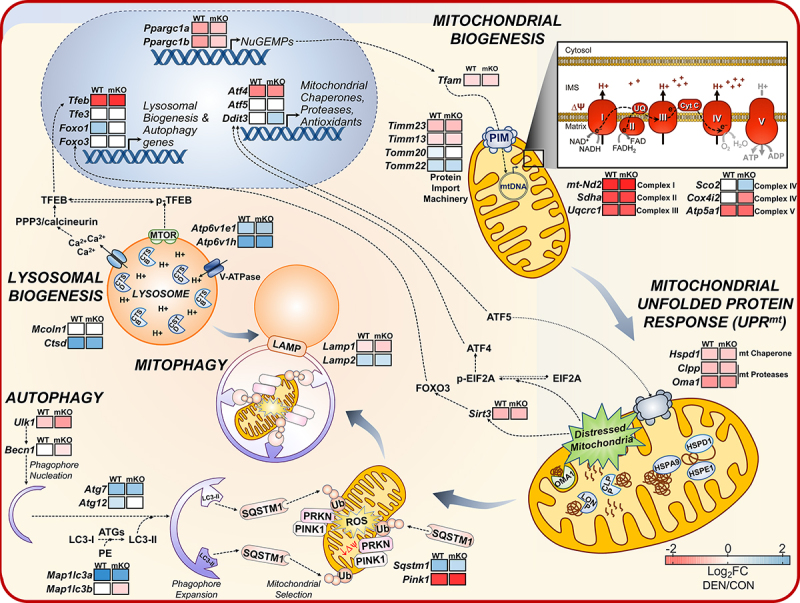
The mitochondrial life cycle in response to 7-day denervation-induced muscle disuse in wild-type (WT) and *tp53* muscle-specific knockout (mKO) animals. Mitochondrial quality control (MQC) is governed by the coordination of multiple signaling pathways, all of which are required for optimal mitochondrial maintenance. Mitochondrial biogenesis describes the process controlling organelle synthesis, whereas the mitochondrial unfolded protein response (UPR^mt^) senses perturbations in the mitochondrial milieu and mounts an appropriate response to either restore mitochondrial homeostasis or promote organelle degradation via mitophagy. Mitophagy involves both lysosomal and autophagy machinery to allow for clearance of dysfunctional organelles. Colored boxes indicate the Denervated/Control Log_2_FC of MQC genes as measured via RNA-Seq and illustrate the discrepancies in gene expression changes with denervation in WT and *tp53* mKO animals, thus highlighting the importance of TP53 in the multifaceted regulation of mitochondrial adaptation to disuse. NuGEMPs, nuclear genes encoding mitochondrial proteins; PIM, protein import machinery.

Together, our data indicate a role for TP53 in regulating mitochondrial function, particularly under stress conditions, and build on previous studies that highlight TP53 as a regulator of mitochondrial remodeling in the presence of another stressor, that of exercise. It remains to be determined what influence TP53 ablation would have at intermediate time points, or in the context of milder disuse conditions. We speculate that under periods of acute inactivity, such as following injury, or during bedrest, TP53 would be important for maintaining mitochondrial content and function until mobility is restored. In addition, as TP53 is involved in exercise-induced mitochondrial biogenesis and remodeling, it would be worthwhile for future studies to examine the influence of TP53 ablation on mitochondrial readaptation following muscle reloading. While this would not be possible following denervation, alternative models such as nerve crush, or tetrodotoxin paralysis could temporarily remove neural signaling, but allow for reinstatement following a period of disuse. In these instances, we think that the absence of TP53 would promote more substantial reductions in mitochondrial quality in muscle during the disuse period, and would result in a significantly blunted ability to restore mitochondrial function following re-innervation, thus leading to exacerbated mitochondrial derangements and further skeletal muscle pathology. From a more clinical perspective, this work also suggests the possibility that patients with cancer, in which TP53 mutations are a common feature, may be more susceptible to disuse-induced muscle mitochondrial defects during treatment or bedrest, in addition to their innate proclivity for cachexia.
